# Race may modify the association between blood type and COVID‐19 infection

**DOI:** 10.1002/jha2.539

**Published:** 2022-07-30

**Authors:** Jiajun Luo, Andrew Craver, Paul Zakin, Liz Stepniak, Kayla Moore, Jaime King, Muhammad G. Kibriya, Julie Johnson, Christopher O. Olopade, Jayant M. Pinto, Karen Kim, Habibul Ahsan, Briseis Aschebrook‐Kilfoy

**Affiliations:** ^1^ Department of Public Health Sciences University of Chicago Chicago Illinois; ^2^ Institute for Population and Precision Health University of Chicago Chicago Illinois; ^3^ Comprehensive Cancer Center University of Chicago Chicago Illinois; ^4^ Center for Research Informatics University of Chicago Chicago Illinois; ^5^ Department of Medicine University of Chicago Chicago Illinois; ^6^ Department of Surgery University of Chicago Chicago Illinois

**Keywords:** blood groups, epidemiology, infection

## Abstract

This study aims to investigate the race/ethnicity‐specific association between blood type and COVID‐19 susceptibility during March, 2020 and December, 2021 using data from the electronic health record at the University of Chicago Medicine. The study population was stratified into four groups: non‐Hispanic White, non‐Hispanic Black, Hispanic, and other. Log‐binomial generalized mixed model was used to estimate the relative risk (RR) and 95% confidence interval (CI). When compared to blood type O, type B was associated with positive COVID‐19 test in Blacks (RR = 1.12, 95% CI: 1.02–1.23), Whites (RR = 1.28, 95% CI: 0.99–1.66), and Hispanic (RR = 1.36, 95% CI: 0.97–1.92).

## 1 INTRODUCTION

Understanding the mechanisms behind racial disparities in COVID‐19 susceptibility and severity is a key scientific priority given the evidence of disproportionate impact in underrepresented communities in the United States, especially for African Americans [1]. While social and economic factors may explain this disparity [2], it remains unknown whether a physiological basis underlies it as well.

Blood type is an established risk factor for many infectious diseases, as blood group can serve as receptors and co‐receptors for pathogens and facilitate intracellular transportation of viral particles [3]. ABO blood type is associated with infection by a variety of viruses including SARS‐CoV‐1 [4, 5, 6, 7]. Although a large body of literature has explored the association between blood type and COVID‐19 susceptibility in the past two years [8, 9, 10], data from underrepresented populations is sparse. Generally, the most consistent finding is that blood type O and Rhesus (Rh)‐negative are associated with lower COVID‐19 susceptibility, but since blood type distribution varies by ancestry [11], whether different populations of different ancestry or race/ethnicities equally benefit from this protection is under investigated [12].

Since 2020, the University of Chicago Medicine (UCM) has conducted more than 100,000 COVID‐19 PCR tests in a highly diverse, heavily burdened, and underrepresented patient population at its main Hyde Park campus. Here, we leverage robust electronic health record (EHR) data to consider the role of ABO and Rh blood type in COVID‐19 risk by race and over time through the pandemic, accounting for major changes to care.

## 2 METHODS

### 2.1 Participants

The study population was restricted to UCM patients aged 18 or older with valid blood type information from March 1, 2020 to December 31, 2021. In total, 31,784 individuals had lab‐verified blood type data in the EHR. We also obtained information on demographics, comorbidities, laboratory results, UCM treatment, and outcomes from the EHR for these patients. Patients who tested multiple times were considered never positive if all tests were negative and ever positive if one or more tests were positive. This study was approved by the University of Chicago Biological Sciences Division Institutional Review Board with a waiver of consent for use of identifiable data.

### 2.2 Measurements

All variables were defined based on information from the UCM EHR (Epic; Epic Systems). COVID‐19 test status was determined by standard PCR in our clinical laboratory. Age, sex, race/ethnicity, and smoking status were also obtained from the EHR. Body mass index (BMI) and comorbidities including hypertension and type 2 diabetes were obtained from most recent EHR information. Blood type was determined using standard clinical laboratory measurements (ABO and Rh types).

### 2.3 Statistical analysis

Log‐binomial generalized mixed model was used to evaluate the relative risk (RR) and 95% confidence interval (CI) for different blood types with type O or Rh‐negative as the references. A random intercept and slope were set for race/ethnicity to control for varying blood type prevalence across racial groups. Additionally, to investigate the association between blood type and COVID‐19 risk among underrepresented groups, we stratified our study population into four racial groups: non‐Hispanic Black, non‐Hispanic White, Hispanic, and other. Moreover, to investigate the related temporal variations, we stratified our study population into two time periods: March to December, 2020 and January to December, 2021. Patients who visited UCM in one period were included in the analysis for that specific period. Therefore, patients who visited UCM in multiple periods were counted in multiple stratified analyses. As vaccination may impact the association between blood type and COVID‐19 risk, we also identified patients who received vaccination at UCM and ran the analysis among these vaccinated population.

All models were adjusted for age, BMI, sex, smoking history, hypertension, and type 2 diabetes. We added a cross‐product term between the racial group and blood type to test the difference between racial groups.

Missing values in the covariates were treated using multiple imputation. A total of 20 complete data sets were generated and used for analyses. Results from the 20 data sets were combined and analyzed [13]. All analyses were performed using R (version 4.1.2).

## 3 RESULTS

Blacks were more likely to test positive for COVID‐19 compared to their White counterparts (Table 1). Patients with hypertension were more likely to test positive in the first year, but not in the following year. Ever positive patients were more likely to have a history of type 2 diabetes. Compared to the White patients, Black patients had a higher prevalence of blood type B, and lower prevalence of type A and Rh negative (Table 2).

**TABLE 1:** Distribution of COVID‐19 tested cohort at UCM by key demographic factors and comorbidities over time

	All time period		March to December, 2020		January to December, 2021	
	Never positive (*n* = 28,527)	Ever positive (*n* = 3257)	Never positive (*n* = 20,877)	Ever positive (*n* = 1679)	Never positive (*n* = 24,873)	Ever positive (*n* = 1626)
Age						
≤35	9052 (31.7)	1009 (31.0)	6404 (30.7)	447 (26.6)	7563 (30.4)	577 (35.5)
35–50	5125 (18.0)	655 (20.1)	3783 (18.1)	315 (18.8)	4609 (18.5)	350 (21.5)
50–65	6559 (23.0)	738 (22.7)	4756 (22.8)	404 (24.1)	5872 (23.6)	350 (21.5)
>65	7791 (27.3)	855 (26.3)	5934 (28.4)	513 (30.6)	6829 (27.5)	349 (21.5)
BMI						
<18.5	830 (2.9)	90 (2.8)	577 (2.8)	42 (2.5)	689 (2.8)	51 (3.1)
18.5–25	8344 (29.3)	795 (24.4)	6036 (28.9)	399 (23.8)	7059 (28.4)	410 (25.2)
25–30	8047 (28.2)	871 (26.7)	5921 (28.4)	437 (26.0)	7028 (28.3)	444 (27.3)
30–40	8177 (28.7)	1031 (31.7)	6121 (29.3)	544 (32.4)	7366 (29.6)	501 (30.8)
>40	2544 (8.9)	392 (12.0)	1933 (9.3)	217 (12.9)	2302 (9.3)	182 (11.2)
Missing	585 (2.1)	78 (2.4)	289 (1.4)	40 (2.4)	429 (1.7)	38 (2.3)
Race						
Non‐Hispanic Black	16,700 (58.5)	2414 (74.1)	12,322 (59.0)	1225 (73.0)	14,444 (58.1)	1226 (75.4)
Non‐Hispanic White	8082 (28.3)	483 (14.8)	6030 (28.9)	241 (14.4)	7224 (29.0)	243 (14.9)
Hispanic	1868 (6.6)	220 (6.8)	1264 (6.1)	134 (8.0)	1658 (6.7)	96 (5.9)
Other	1877 (6.6)	140 (4.3)	1261 (6.1)	79 (4.7)	1547 (6.2)	61 (3.8)
Sex						
Female	16,869 (59.1)	2017 (61.9)	12,869 (61.6)	1008 (60.0)	14,983 (60.2)	1039 (63.9)
Male	11,658 (40.9)	1240 (38.1)	8008 (38.4)	671 (40.0)	9890 (39.8)	587 (36.1)
Smoking						
Never	13,678 (48.0)	1613 (49.5)	10,425 (49.9)	829 (49.4)	12,398 (49.9)	814 (50.1)
Active	3598 (12.6)	302 (9.3)	2505 (12.0)	146 (8.7)	3066 (12.3)	164 (10.1)
Quit	7352 (25.8)	893 (27.4)	5888 (28.2)	476 (28.4)	6767 (27.2)	426 (26.2)
Missing	3899 (13.7)	449 (13.8)	2059 (9.9)	228 (13.6)	2642 (10.6)	222 (13.7)
Hypertension						
No	24,040 (84.3)	2663 (81.8)	17,797 (85.3)	1350 (80.4)	20,953 (84.2)	1360 (83.6)
Yes	4482 (15.7)	594 (18.2)	3080 (14.8)	329 (19.6)	3915 (15.7)	266 (16.4)
Missing	5 (0)	0 (0)	0 (0)	0 (0)	5 (0)	0 (0)
Diabetes						
No	25,735 (90.2)	2772 (85.1)	18,950 (90.8)	1417 (84.4)	22,447 (90.3)	1402 (86.2)
Yes	2787 (9.8)	485 (14.9)	1927 (9.2)	262 (15.6)	2421 (9.7)	224 (13.8)
Missing	5 (0)	0 (0)	0 (0)	0 (0)	5 (0)	0 (0)
ABO blood type						
O	13,469 (47.2)	1492 (45.8)	9776 (46.8)	759 (45.2)	11,710 (47.1)	753 (46.3)
A	8715 (30.6)	934 (28.7)	6412 (30.7)	463 (27.6)	7610 (30.6)	479 (29.5)
B	5087 (17.8)	675 (20.7)	3760 (18.0)	377 (22.5)	4427 (17.8)	316 (19.4)
AB	1250 (4.4)	154 (4.7)	926 (4.4)	79 (4.7)	1119 (4.5)	77 (4.7)
Missing	6 (0)	2 (0.1)	3 (0)	1 (0.1)	7 (0)	1 (0.1)
Rh type						
Negative	2535 (8.9)	250 (7.7)	1887 (9.0)	123 (7.3)	2233 (9.0)	131 (8.1)
Positive	25,992 (91.1)	3007 (92.3)	18,990 (91.0)	1556 (92.7)	22,640 (91.0)	1495 (91.9)

**TABLE 2:** Distribution of blood types by racial groups

	Non‐Hispanic Black (*n* = 19,114)	Non‐Hispanic White (*n* = 8565)	Hispanic (*n* = 2088)	Other (*n* = 1883)
ABO blood type				
O	9351 (48.9)	3530 (41.2)	1162 (55.7)	918 (45.5)
A	4956 (25.9)	3474 (40.6)	636 (30.5)	583 (28.9)
B	3986 (20.9)	1138 (13.3)	233 (11.2)	405 (20.1)
AB	815 (4.3)	421 (4.9)	57 (2.7)	111 (5.5)
Missing	6 (0)	2 (0)	0 (0)	0 (0)
Rh type				
Negative	1185 (6.2)	1353 (15.8)	113 (5.4)	134 (6.6)
Positive	17,929 (93.8)	7212 (84.2)	1975 (94.6)	1883 (93.4)

**FIGURE 1:**
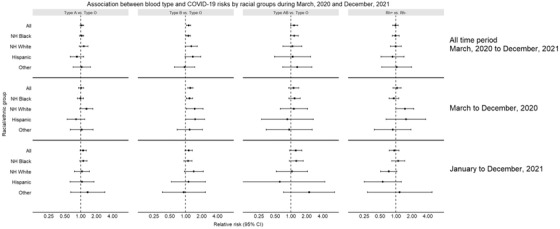
Association between blood type and COVID‐19 risk by race and over time

Overall, the association was significant for blood type B (RR = 1.14, 95% CI: 1.04–1.24) and borderline significant for type AB (RR = 1.15, 95% CI: 0.99–1.35), compared to type O. When stratified by race/ethnicity, significant association with type B was observed among Black patients (RR = 1.12, 95% CI: 1.02–1.23), but only borderline significant among White (RR = 1.28, 95% CI: 0.99–1.66) and Hispanic (RR = 1.36, 95% CI: 0.97–1.92) patients (Figure 1).

When stratified by time period, the significant association was only observed between March and December, 2020. In this time period, compared to type O, type B was significantly association with COVID‐19 risk in all racial groups except for other race (all: RR = 1.22, 95% CI: 1.09–1.37; Black: RR = 1.18, 95% CI: 1.03–1.35; White: RR = 1.48, 95% CI: 1.03–2.14; Hispanic: RR = 1.51, 95% CI: 1.01–2.26) (Figure 1). The differences in the association magnitude between Black and White patients, and between Black and Hispanic patients, had a *p* value of 0.06 and 0.05, respectively. Moreover, blood type A was also borderline associated with COVID‐19 risk among White patients (RR = 1.29, 95% CI: 0.97–1.71). In the second year, though some suggestive associations were observed, these associations were not significant.

Rh blood type was observed to be associated with COVID‐19 risk among White patients in the first time period (RR = 1.48, 95% CI: 1.00–2.17), while not among other groups or in the other time period.

Among the vaccinated population, no association was observed between any blood type and COVID‐19 risk (Table 3).

**TABLE 3:** Association between blood type and COVID‐19 risk among immunized population

	All immunized population		Immunized non‐Hispanic Black		Immunized non‐Hispanic White	
	*N* *	OR (95% CI)	*N* *	OR (95% CI)	*N* *	OR (95% CI)
ABO blood type						
O	673/6021	Ref	481/3098	Ref	114/2064	Ref
A	444/4112	1.08 (0.97–1.21)	269/1642	1.05 (0.92–1.21)	115/1968	1.06 (0.82–1.36)
B	290/2236	1.10 (0.97–1.26)	216/1283	1.07 (0.92–1.24)	43/665	1.15 (0.82–1.62)
AB	66/600	1.05 (0.83–1.33)	47/278	1.07 (0.81–1.41)	11/245	0.80 (0.44–1.47)
Rh type						
Negative	119/1278	Ref	63/384	Ref	43/786	Ref
Positive	1355/11,693	0.99 (0.83–1.18)	951/5919	0.99 (0.78–1.25)	240/4156	1.06 (0.77–1.45)

OR, odds ratio; CI, confidence interval.

*Note*: No results shown for immunized Hispanic and other race groups due to limited sample size.

* The number of ever positive/never positive.

## 4 DISCUSSION

In general, COVID‐19 susceptibility varied by ABO blood type, especially in 2020. Specifically, type B was associated with increased risk for viral infection among all racial groups; type AB was associated with increased infection risk among Blacks, but not other racial groups. A significant association was also observed for Rh blood type among Whites in 2020.

Findings from the study suggested a modifying role of race/ethnicity in COVID‐19 [14, 15]. Some possible differences were observed in the associations between blood type and COVID‐19 risks. For example, the association of blood type B was more pronounced in Whites and Hispanic patients than in Black patients. In addition to currently unknown biological mechanisms, this difference may also be explained by the varying prevalence across racial groups: the prevalence of blood type B was lower in the Black patients in the study population. We also observed suggestive association of blood type A among White patients, but not Black patients. These findings were consistent with an earlier study that reported associations between blood type A and respiratory distress syndrome among White patients, but not in Black patients [15]. However, given the uncertainty, more studies are warranted to investigate the racial differences.

We only observed associations with ABO and Rh blood types in 2020, consistent with early studies from the same period [8, 9, 10]. Several possible reasons may explain the null association in the later periods. First, the variants evolved, becoming less sensitive to blood types. Second, preventive measures adopted in the later periods mitigated the impact of blood type. Third, the availability of pharmacologic/biologic therapies or vaccines after the early stage of the pandemic protected patients against any adverse association with blood types.

Several limitations should be considered when interpreting our results. First, our results are based on data collected through COVID‐19 testing during the pandemic, which may result in selection bias. However, the distribution of blood types in our sample is similar to the general population, so selection bias should be minimal as related to blood type. Second, we lacked data on behavioral or occupational differences during the pandemic, which all might vary by race/ethnicity.

In conclusion, race/ethnicity may modify the association between blood type and COVID‐19 susceptibility. Although types A and B were observed to be associated with COVID‐19 susceptibility, these associations were stronger in Whites than in Blacks and the protection was mitigated significantly as the pandemic context evolved. Identifying biologic mechanisms that underlie these results could help uncover strategies to prevent or treat this major disease.

## CONFLICT OF INTEREST

The authors declare they have no conflicts of interest.

## ETHIC STATEMENT

This study was approved by the University of Chicago Biological Sciences Division Institutional Review Board with a waiver of consent for use of identifiable data. It was determined that this analysis could not be reliably executed without the use of identifiable data and that it was impractical to obtain consent from all subjects.
